# The effect of the ongoing civil strife on key immunisation outcomes in the North West and South West regions of Cameroon

**DOI:** 10.1186/s13031-021-00341-0

**Published:** 2021-02-10

**Authors:** Yauba Saidu, Marius Vouking, Andreas Ateke Njoh, Hassan Ben Bachire, Calvin Tonga, Roberts Mofor, Christain Bayiha, Leonard Ewane, Chebo Cornelius, Ndi Daniel Daddy Mbida, Messang Blandine Abizou, Victor Mbome Njie, Divine Nzuobontane

**Affiliations:** 1 Clinton Health Access Initiative, Cameroon Country Office, Third floor, Y-building, Rue 1775, Nouvelle Route Bastos, Yaoundé, Cameroon; 2 Institute for Global Health, University of Siena, Siena, Italy; 3 Central Technical Group, Expanded Programme on Immunisation, Ministry of Public Health, Yaoundé, Cameroon; 4 Center for the Development of Best Practices in Health, Central Hospital Yaoundé, Ministry of Public Health, Yaoundé, Cameroon; 5 Regional Technical Group, Expanded Programme on Immunisation, South West Regional Delegation for Public Health, Buea, Cameroon; 6 Faculty of Science, University of Buea, Buea, Cameroon; 7 Regional Technical Group, Expanded Programme on Immunisation, North West Regional Delegation for Public Health, Bamenda, Cameroon; 8 Ministry of Public Health, Yaoundé, Cameroon; 9 Department of Public Health, University of Buea, Buea, Cameroon

**Keywords:** Immunisation coverage, Civil strife, Conflict, Anglophone crises, Cameroon

## Abstract

**Background:**

Civil strife has long been recognized as a significant barrier in the fight against vaccine preventable diseases in several parts of the world. However, little is known about the impact of the ongoing civil strife on the immunisation system in the Northwest (NW) and Southwest (SW) regions of Cameroon, which erupted in late 2016. In this paper, we assessed the effect of the conflict on key immunisation outcomes in the North West and South West regions of Cameroon.

**Methods:**

Data were obtained from the standard EPI data reporting tool, the District Vaccine and Data Management Tool (DVDMT), from all the districts in the two regions. Completed forms were then reviewed for accuracy prior to data entry at central level. Summary statistics were used to estimate the variables of interest for each region for the years 2016 (pre-conflict) and 2019 (during conflict).

**Results:**

In the two regions, the security situation has deteriorated in almost all districts, which in turn has disrupted basic healthcare delivery in those areas. A total of 26 facilities were destroyed and 11 healthcare workers killed in both regions. Reported immunisation coverage rates for key antigens including, BCG, DPT-3 and MR, witnessed a dramatic decline between 2016 and 2019, ranging from 22% points decline for BCG in the NW and to 42% points decline for DPT-3 in the SW. Similarly, the proportion of districts with DPT-3 coverage of at least 80% dropped from 75% in 2016 to 11% in 2019 in the NW. In the SW this proportion dropped from 16% in 2016 to 0 % in 2019.

**Conclusion:**

Our data demonstrates the marked negative impact of the ongoing civil strife on key immunisation outcomes in the two regions and the country at large. This decline could amplify the risk of vaccine preventable diseases vaccine preventable diseases outbreaks in the two regions. Besides the ongoing actions to contain the crises, effective strategies for reaching children in the conflict zones as well as the internally displaced population are needed. There is also the need to rebuild destroyed facilities as well as to protect health facilities and staff from targeted violence.

## Introduction

There is increasing recognition from the global health community that the goal of equitably extending the benefits of immunisation to all infants is far from being achieved [1]. One underlying reason for this is that a sizable proportion of infants reside in areas with civil strife. In 2017, for instance, nearly 8 million unimmunized infants resided in areas with civil strife [2], where a sizable number of them died from diseases that could have been easily and affordably prevented by vaccines [3, 4].

The death toll is often high because civil strife habitually creates conditions that increase susceptibility to VPDs, while also damaging immunization systems. Civil strife can lead to mass displacement of people, overcrowding, and limited access to food, water, hygiene and sanitation services [5], which invariably amplify the spread of, and mortality from, vaccine preventable diseases (VPD) [6, 7]. For example, overcrowding may increase the risk of measles and pneumonia while restricted food access can result in malnutrition, which in turn may increase susceptibility to VPD [8] as well as limit the generation of robust immune responses after vaccination [9]. In addition, civil strife can also cripple immunisation systems through the destruction of buildings, decimation of staff, and disruption of supply lines and service delivery, which in turn can lead to a sizable decline in immunisation programme performance [10]. In Afghanistan, for instance, a marked deterioration of immunisation system was noted following the onset of armed conflict in 2001 [4]. Similar deteriorations, which eventually triggered outbreaks of polio, have been reported from Chad [11], Central African Republic [12], Pakistan [13], South Sudan [14], Somalia [15] and Ukraine [16] amongst others. In almost all of these countries, onset of armed conflicts resulted into a sudden drop in vaccination coverage, with some countries registering national coverages below 50% [17].

Although the devastating effect of conflicts on the immunisation system has been documented in many countries, little is known about the influence of the ongoing civil strife on Cameroon’s immunisation system. Cameroon has enjoyed decades of peace until late 2016 when armed violence erupted in the NW and SW regions [18]. The conflict is characterized by fierce fighting between several separatist militia groups and government forces [19]. The security situation in almost all health districts in these regions has been extremely delicate and access to healthcare services has become a major concern. The protracted nature of the conflict has adversely affected the immunisation programme and despite efforts at various levels, provision of immunisation services to those who need it has become extremely challenging. In this paper, we assess the impact of this conflict on various immunisation outcomes in the two regions.

## Methods

### Study settings

The study was conducted in Cameroon, which is politically divided into ten regions: eight of which are French speaking and the remaining two are English speaking. This dichotomy can be traced back to World War I, when France and Britain joined forces to attack and seize Cameroon, which until then was a German colony. Following the seizure, the German colony was split into two entities and 83% of the territory was awarded to France by the League of Nation and the remaining 17% to Britain [20]. Each of these countries then influenced its mandated territory with its language and culture and eventually, these territories evolved into Francophone and Anglophone zones. Currently, there are two Anglophone regions - the NW and SW regions - which are home to nearly 5 million people, with diverse cultural and traditional backgrounds. Over 44% percent of this population reside in rural areas [20], where the main economic activities are farming and fishing [20].

### Organisation of immunisation system in the North West and South West regions

Both regions are divided into Health Districts (HD), which in turn are divided into health areas. Each health area has a leading health facility, which is generally public, but can be private in some health areas. The leading facility coordinates the distribution of vaccines and supplies and reports coverage rates for all facilities, both public and private, within the health area. Prior to the crises, the SW had 18 functional health districts, and 308 health facilities, of which 255 (83%) were offering immunisation services [21]. The North West region, on the other hand, had 19 functional districts, and 400 health facilities, of which 351 (88%) were offering immunisation. These facilities obtain vaccines and other immunisation supplies from the districts on a monthly basis via a pull mechanism and deliver them to the target population according to the following schedule:

One dose of Bacillus Calmette-Guerin (BCG) at birth or on first contact by a health worker, three doses of diphtheria, pertussis and tetanus containing vaccines (DPT1, DPT2 and DPT3) and 13-pneumococal conjugate vaccine (PCV-13) at 6, 10 and 14 week, four doses of oral polio vaccine (OPV0, OPV1, OPV2 and OPV3) at birth, 6, 10 and 14 weeks, two doses of rotavirus vaccine at 6 and 10 weeks, one dose of inactivated polio vaccine (IPV) at 14 weeks, and one dose of yellow fever and combined measles and rubella vaccine (MR) at 9 months (Table 1). According to national guidelines, these vaccines are administered to all eligible infants at the facility on daily basis and in outreach sites, where feasible [22].

**Table 1:**
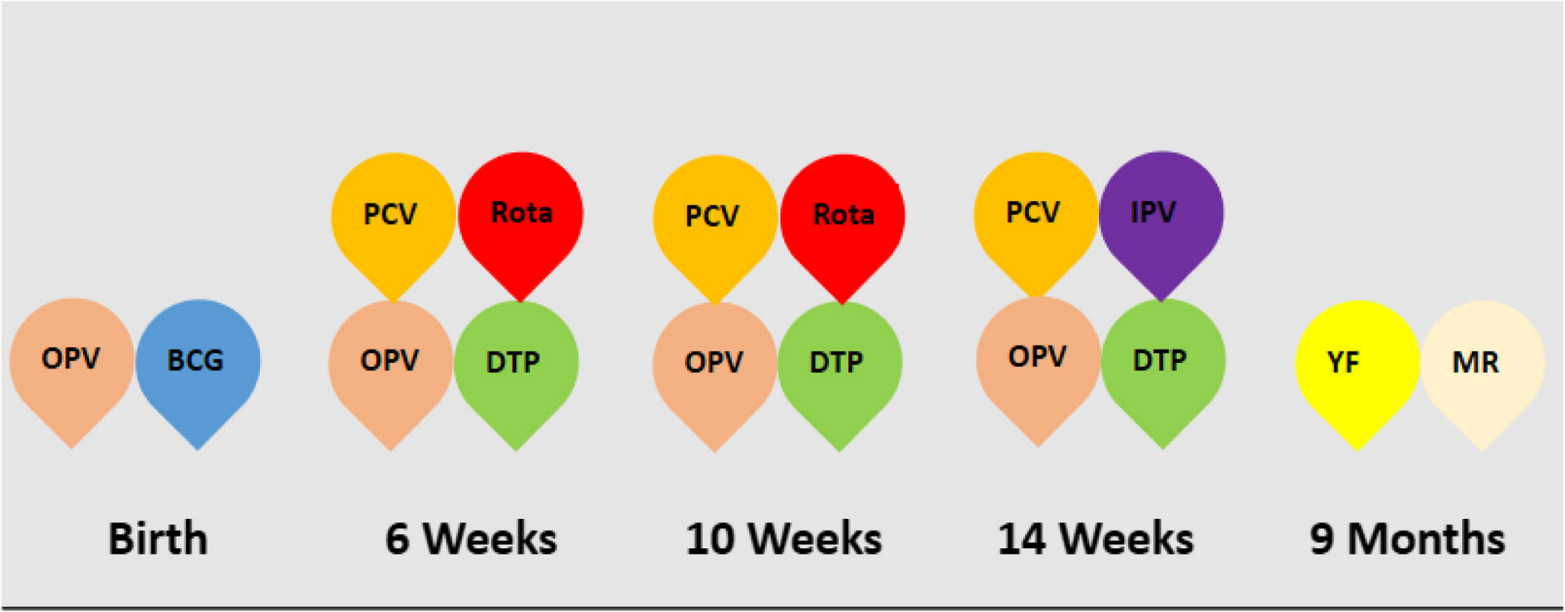
Immunization schedule in Cameroon. Illustrates the vaccination calendar of Cameroon. Each color is assigned to a particular vaccine. The timeframe below each group of vaccines indicates the age from birth at which the vacciness are authorized to be administered to the target

### Data sources

The data for this study were retrieved from the standard EPI data reporting tool, the District Vaccine and Data Management Tool (DVDMT). According to standard practice, this tool is updated on a monthly basis with data from health facilities in each district. Regional EPI heads used a predefined questionnaire to abstract key variables, including the security profile of the health districts, service delivery, vaccine logistics, and others. These data were extracted for 2016, when the conflict first started, and 2019, after more than 2 years of continued conflict, in order to evaluate the impact of the conflict on these variables. Following data collection, the regional EPI heads returned the completed forms to the central level, where they were screened for accuracy and completeness before data entry. We resolved any identified data discrepancy by directly calling the regional head or the district medical officer. We then analyzed the data using Excel 2016 and used summary statistics to estimate the variables of interest individually for the years 2016 and 2019.

## Results

### Evolution of the crises over time

**Fig. 1:**
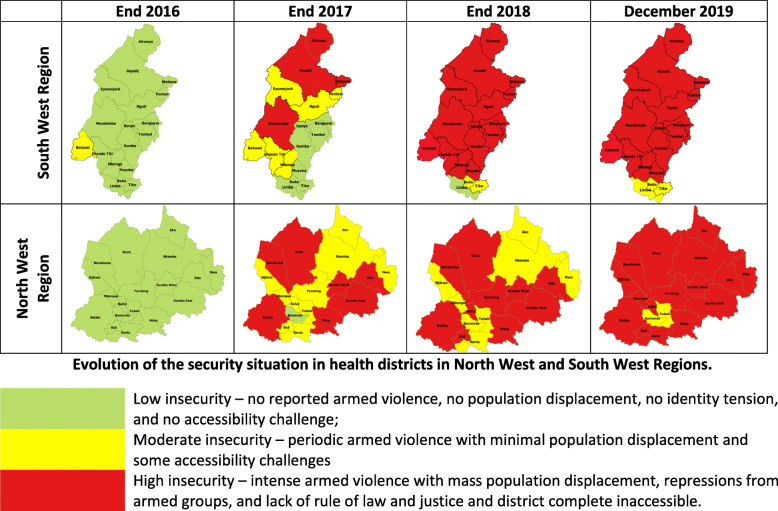
Evolution of the security situation in health districts in North West and South West Regions. A schematic representation of the evolution of the security profile of the northwest and southwest regions of Cameroon from 2016 to December, 2019. For the respective years, zones in green represent health districts considered not to experience armed violence. Zones in yellow represent health districts with periodic armed violence associated with some accessibility challenges. Red illustrates health districts with full blown armed conflict and zones are completely inaccessible

Figure 1 shows the evolution of the security situation in each region across the study period. As illustrated, nearly all health districts were completely safe and accessible by the end of 2016. The only exception was the Bakassi Health District in the SW region, which was considered at the time as a district with moderate insecurity because of the periodic armed violence perpetrated by armed groups in this area, unrelated to the Anglophone crises. This armed violence originated after Nigeria effectively pulled out its military from the area following the Greentree Agreement that finally resolved the Cameroon-Nigeria Bakassi Peninsula dispute that erupted after the discovery of the rich deposits of oil/gas reserves in the early 1990s [23].. Like the Bakassi Health District, the security situation in both regions began degenerating in the first quarter of 2017 and by the end of 2018, 17 of the 18 districts in the SW region and all the 19 districts in the NW region were highly insecure as a result of a full blown armed conflicts in these districts.

### Impact of the crises on the coverage of key antigens

**Fig. 2:**
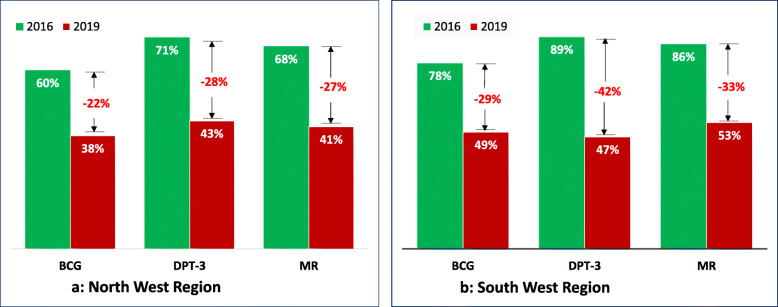
Evolution of vaccination coverage in the two regions with civil strife. **a** Shows the percentage of the total vaccination coverage for each of the vaccines (BCG, DTC-3 and MR) for the North West region in 2016 and 2019. The bars color corresponds to the year whose coverage is represented for each of the vaccines presented. The gap whose value is represented in red indicates the absolute difference between the 2016 and 2019 vaccination coverages for each of the vaccines. **b** Shows the percentage of the total vaccination coverage for each of the vaccines (BCG, DTC-3 and MR) for the South West region in 2016 and 2019. The bars color corresponds to the year whose coverage is represented for each of the vaccines presented. The gap whose value is represented in red indicates the absolute difference between the 2016 and 2019 vaccination coverages for each of the vaccines

Figure 2 shows the evolution of reported immunisation coverage rates for three antigens (BCG, DPT-3 and MR) in the two conflict regions. As illustrated, coverage rates for these antigens witnessed a dramatic decline between 2016 and 2019, ranging from 22 percentage-point (pp) decline for BCG in the NW region to a 42 pp. decline for DPT-3 in the SW region. A similar trend was observed for other antigens not shown in Fig. 2, including PCV-13, Rotarix-2 and OPV. For instance, in the NW region, coverage rate for PCV-13 dropped by 28 pp., falling from 71% in 2016 to 43% in 2019; similarly, coverage for IPV dropped by 27 pp., plummeting from 70 to 43%. In the SW region, coverage for PCV-13 declined from 88 to 47% (41 pp); IPV dropped from 87 to 47% (40 pp) and Rotarix-2 from 87 to 49% (38 pp).

### Impact of the crises on geographic equity of immunisation in the two regions

Table 2 shows the comparison of the immunisation performance of districts between 2016 and 2019. As can be seen, in 2016, over 75% of districts in the NW region had coverages rates for BCG, DPT-3 and MR that were over 80%. In 2019, however, just about 11% of districts in this region reached 80% coverage for these antigens. Indeed, about 70% of districts had coverage rates below 40%. Worse still, 39% districts registered coverages lower than 20%. A somewhat similar trend was observed for districts in the SW region, though their performance in 2016 was comparatively lower than their NW counterparts. It is worth noting that few districts (< 16%) in the SW region had coverages above 80% in 2016. In 2019, no district reached the 80% coverage mark for any of the antigens. In contrast, over 50% of districts had a coverage below 50% for all antigens.

**Table 2:**
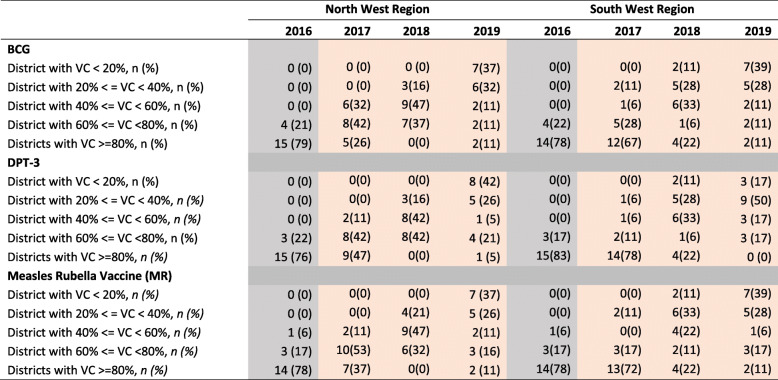
Trend in vaccine coverage (VC) in the two conflict regions

Presents the evolution in the vaccination coverage in all the health districts of the northwest and southwest per year from 2016 to 2019 for DPT-3, BCG and MR. The absolute numbers (n) indicate the number of districts per region whose coverage fall within the respective coverage ranges for each of the vaccines. In brackets are the percentages obtained from the denominator of 19 health districts for the northwest and 18 for the southwest*BCG* Bacillus Calmette-Guerin, *DPT-3* Third dose of Diphtheria, Pertussis and Tetanus containing vaccines, *MR* Measles Rubella Vaccine, *VC* Vaccination Coverage

### Impact of the crises on selected immunisation service variables

**Fig. 3:**
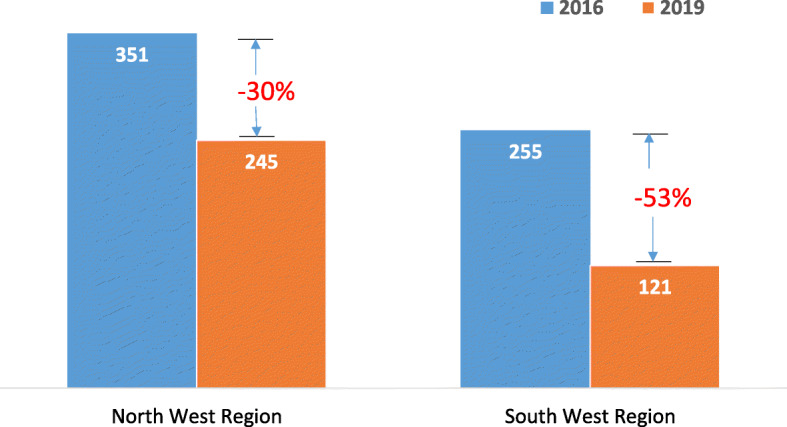
Effect of crises on vaccinating health facilities. The drop in the total number of vaccinating health facilities in the north west region and south west region between 2016 and 2019. The color of each bar corresponds to the year whose number of functional health facilities are represented by the bar. The gap whose value is represented in red indicates the percentage drop in the number of functional health facilities for the respective regions between 2016 and 2019. This gap is obtained by dividing the number of vaccinating health facilities that were no longer functional after 2016 by the number of functional vaccinating health facilities in the respective regions in 2016

Figure 3 presents the effect of the conflict on functional status of vaccinating health facilities. As can be seen, the number of facilities offering immunisation in both regions declined dramatically between 2016 and 2019, falling by 30 and 53% in the NW and SW respectively. In total, 26 facilities have been destroyed and all of these occurred in the SW region. Furthermore, 128 facilities in the SW and 106 in the NW have been permanently shut down because of the rising level of insecurity. To date, four healthcare workers in NW and seven in the SW have been brutally assassinated, which has caused other healthcare staff to flee from their duty posts to safer locations in neighboring communities or regions. Facility shutdown and decimation and fleeing of health personnel have also affected other critical indicators such as vaccine distribution and transmission of standard EPI reports. Indeed, by the end of November 2019, just about 10% of facilities in the NW and 7% in the SW regions transmitted their monthly reports.

Similar declines were observed with immunisation service delivery. In the NW, the proportion of fixed-post immunisation sessions dropped by 27%, falling from 8947 sessions in 2016 to 6523 sessions in 2019. Similarly, the proportion of outreach sessions declined by 75%, falling from 8362 sessions in 2016 to 2092 sessions in 2019. In the SW, the proportion of fixed-post session dropped by 46%, decreasing from 8952 sessions in 2016 to 4808 sessions in 2019. Likewise, the proportion of outreach sessions declined by 65%, dropping from 3657 sessions in 2016 to 1292 sessions in 2019.

## Discussion

The consequences of the ongoing civil strife in the NW and SW regions of Cameroon (or the “Anglophone crises”) have been enormous. Over 3000 people have been killed in direct fighting or died from war related conditions such as gunshot wounds, starvation or poor hygienic conditions [24]. In addition, over half a million people (the majority of whom are women and children) have been displaced by the conflict, with 35,000 fleeing to neighboring Nigeria [25]. In addition to these human catastrophes, the conflict has had a significant impact on the infrastructure needed for the delivery of healthcare services, including immunisation in the two regions.

The goal of this study was, therefore, to assess the effect of the conflict on the immunisation system in these two regions, with particular focus on the effect on coverage for key antigens and other variables. Overall, we found a dramatic decline in coverage for all antigens in both regions. For instance, between 2016 and 2019, coverage for DPT-3 dropped by 28 and 42 points in the NW and SW respectively. A similar trend was observed for antigens against measles, poliomyelitis, pneumococcal and rotavirus (Fig. 2). The effect of the conflict on geographic equity has also been devastating. Indeed, prior to the onset of the conflict, all districts in both regions had acceptable immunisation coverages for all antigens, except for three extremely enclaved rural districts in the SW (Bakassi, Konye, and Wabane). By November 2019, over 60% of districts in both regions had coverages below 50% for nearly all antigens, with a sizable number of districts having coverages below 20%. This situation, which appears to be worsening, has also had a profound effect on national coverage and equity. Indeed, during the same period, national coverage for DPT-3 gradually declined from 84% in 2016 to 75% in 2019 and the proportion of districts with a coverage surpassing 80% dropped from 60% in 2016 to 45% in 2019 [26]. This dramatic decline in coverage and equity as the conflict progresses is consistent with findings from several war torn countries, including Afghanistan [4], Democratic Republic of Congo [27], Syria [28] and Yemen [29] amongst others.

The observed decline in coverage and equity can be attributed to the direct effect of the conflict on the immunisation system. Indeed, the number of facilities offering immunisation services have dramatically declined as the conflict intensifies. For example, by November 2019, the number of facilities offering immunisation in the NW and SW declined by 53 and 30% respectively. This decline was a direct consequence of facilities closing down because of the rising level of insecurity, which caused many healthcare workers to flee from their duty stations. In a handful of cases, facilities were openly burnt down, destroyed, or looted by arm groups [30]. The functioning health facilities, on the other hand, are confronted with complex realities, including the prevailing security challenge, which has remarkably impacted all basic immunisation service delivery like replenishing of basic immunisation inputs, as well as planning and conducting fix and outreach immunisation sessions. In addition, fierce fighting in many areas has instilled fear in parents, rendering them hesitant to take their infants to health facilities or outreach sites for vaccination. Collectively, these factors have led to a decline in immunisations services in both regions. In our study, we found that fix post immunisation sessions have declined by roughly 46 and 27% in SW and NW respectively and similar trends were observed with outreach immunisations. The later decline is problematic because outreaches account for about 30% of all immunisations in both regions [26]. With a quasi-cessation of this strategy in both regions, almost 21,730 children will be left unimmunized and thus unprotected against VPD.

The challenge of immunizing infants from these regions extends beyond reaching infants in the conflict regions. As mentioned previously, the conflict has created a mass of internally displaced people, the majority of whom are women and children. Although official reports indicate that half a million people from these regions have fled to the neighboring regions, there is limited clarity on the specific destination of these population sub-groups. This leaves open the question on the geographic denominator to which they will now belong. It is highly possible that these displaced populations are not being adequately included in population denominators in their new locations, which in turn would mean that coverage figures in other regions could actually be lower than currently reported. This challenge highlights the need to sensitize facilities and healthcare workers in districts where these populations are likely to be found so that these displaced individuals and their children are properly captured into the health system in their new locations.

In our study, the reported violence against health facilities and health staff has been devastating. Burning down, destroying, and looting of facilities, coupled with fatal attacks on health staff, undermine the effectiveness of life-savings public health programmes, including immunisation. As seen in this conflict, these actions clearly disrupt service delivery and engender a demoralized workforce. Although there is a global consensus on “zero tolerance” of violence against health facilities and workforce during conflicts [31], mechanisms to translate this global principle into practice seem to be limited in our setting. With basically all funding diverted to contain the security situation, limited resources are available to apply the WHO guideline on making “hospitals safe in emergencies” [32]. There is a need for all stakeholders, including government and non-governmental organisations as well as community and religious leaders, to emphasize on the protection of facilities and health staff to counteract any negative impact on the immunization system. This momentum is necessary to ensure continuous provision of essential health care services to the populations of these two regions. By so doing, we pay homage to the courageous health workers who lost their lives serving the needy, while also instilling hope in an already despaired population. Restoring hope among the population will also require crafting strategies and mobilizing the necessary resources to immunize the un-immunized in the conflict zones as well as the internally displaced. It will also require mobilizing the necessary stakeholders and resources to rebuild the destroyed society.

To our knowledge, this study is the first to systematically report the impact of the ongoing civil strife on immunisation systems in the NW and SW regions of Cameroon. But despite this merit, there are several limitations of this study that are worth highlighting. First, the primary data for this study were collected under volatile conditions, which could have introduced bias into the data, especially the data for 2019. Secondly, the primary denominator, which was used by districts to calculate coverage data for 2019, was assigned by the central level and the denominator was based on projections from the 2005 national census. Using centrally assigned denominators may likely result in inaccurate coverage estates for the districts in these regions, primarily because of massive migration of people from these regions. Third, the regions have suffered from prolonged internet and transport shutdowns, which invariably affected data transmission to higher levels. This later challenge suggests that the data from the two regions were often incomplete most of the time. Notwithstanding, we strongly believe that our study sets the scene for preliminary understanding of the impact of the ongoing crises on key immunisation outcomes and child health in general, in these affected regions. We hope that our findings will stimulate future research needed to document the vaccination status of infants in the crises zones as well as that of internally displaced infants. In addition, further research will be needed to establish effective strategies for reaching children as well as determine appropriate actions to prevent violence against health facilities and health care workers.

## Conclusion

Our study findings suggest a considerable negative effect of the Anglophone crises in the North West and South West Regions of Cameroon on immunization outcomes. The dramatic decline in coverage and equity between 2016 and 2019 suggests that many infants in these regions remain susceptible to VPD. The sharp decline in coverage and equity seems to be a direct consequence of the armed conflict on the healthcare system, including disruption of supply lines, destruction of facilities and decimation of healthcare workers. The prevailing situation in these regions has also affected the national coverage for all antigens, which have also witnessed a sizable decline. This situation is already putting the entire country at risk of VPD outbreaks, as evident by the ongoing massive measles outbreak. Our results humbly represent a call to action to rapidly immunize children from these regions, both those residing in conflict zones and the internally displaced. In the short term, there is the need to identify effective strategies and mobilize resources for reaching these children and protecting health facilities and health workers from violence. In the long term, there is the need to rebuild the infrastructure that was destroyed and to re-establish the systems and the supply lines that have been disrupted. Above all, peace needs to return to these regions to ensure permanent improvement in immunization coverage and prevention of deaths from vaccine-preventable diseases.

## Data Availability

The datasets used and/or analysed during the current study are available from the corresponding author on reasonable request.

## Abbreviations

BCG: Bacille, Calmette Guerin
DVDMT: District Vaccine and Data Management Tool
DPT: Diphtheria, Pertussis and Tetanus
HD: Health District
IPV: Inactivated Polio Vaccine
MR: Measles and Rubella
NW: North West
OPV: Oral Polio Vaccine
PCV: Pneumococcal Conjugate Vaccine
SW: South West
VPD: Vaccine Preventable Diseases

## References

[1] World Health Organisation. Assessment Report of the Global Vaccine Action Plan,” no. September, 2018.” [Online] 2018. Available: https://www.who.int/immunization/global_vaccine_action_plan/SAGE_GVAP_Assessment_Report_2018_EN.pdf. (Accessed: 18 Jan 2019).

[2] VanderEnde K, Gacic-Dobo M, Diallo MS, Conklin LM, Wallace AS (2018). Global routine vaccination coverage — 2017. MMWR Morb Mortal Wkly Rep.

[3] “UNICEF (2019). Immunisation and Conflict.” [Online]. Available: https://www.unicef.org/immunisation/immunisation-and-conflict. (Accessed: 18 Jan 2019.

[4] Taufiq M, Keiko N, Masashi K, Kaoruko S, Takehito T (2007). Impact of conflict on infant immunisation coverage in Afghanistan : a countrywide study 2000–2003.

[5] Salama P, Spiegel P, Talley L (2004). Lessens learned from complex emergencies over past decade. Lancet.

[6] Nnadi C, Etsano A, Uba B, et al. Approaches to Vaccination Among Populations in Areas of Conflict. J Infect Dis. 2017;216(suppl_1):S368-S372. 10.1093/infdis/jix175. 10.1093/infdis/jix175 PMC5754212 28838202

[7] Close RM, Pearson C, Cohn J (2016). Vaccine-preventable disease and the under-utilisation of immunisations in complex humanitarian emergencies. Vaccine.

[8] Caulfield LE, de Onis M, Blössner M, Black RE. Undernutrition as an underlying cause of child deaths associated with diarrhea, pneumonia, malaria, and measles. Am J Clin Nutr. 2004;80(1):193–8. 10.1093/ajcn/80.1.193. 10.1093/ajcn/80.1.193 15213048

[9] Jones KD, Berkley JA (2014). Severe acute malnutrition and infection. Paediatr Int Child Health.

[10] Connolly MA, Gayer M, Ryan MJ, Salama P, Spiegel P, Heymann DL (2004). Communicable diseases in complex emergencies: impact and challenges. Lancet.

[11] World Health Organisation (WHO). Poliomyelitis in Chad. WHO website. http://www.who.int/csr/don/2011_06_10a/en/. Accessed 24 Jan 2019.

[12] Gouandjika-Vasilache I, Mazitchi A, Gumede N (2013). Wild poliovirus importation, Central African Republic. Emerg Infect Dis.

[13] END Polio. Polio in Pakistan. END Polio Pakistan website. http://www.endpolio.com.pk/polioin-pakistan. Accessed 21 Jan 2019.

[14] World Health Organisation (WHO). Poliovirus in South Sudan and Madagascar. Disease Outbreak News, November 14, 2014. WHO website.

[15] United Nations Children’s Fund (UNICEF). Polio outbreak in Somalia continues to spread. UNICEF website. https://www.unicef.org/infobycountry/somalia_69874.html. Accessed 24 Jan 2019.

[16] Bagcchi S (2015). Inadequate vaccine coverage fuels polio outbreak in Ukraine. Lancet Infect Dis.

[17] Grundy J, Biggs BA (2019). The impact of conflict on immunisation coverage in 16 countries. Int J Health Policy Manag.

[18] Wikipedia. Anglophone Crisis. https://en.wikipedia.org/wiki/Anglophone_Crisis. Accessed 24 Jan 2019.

[19] British Broadcasting Coperation. Cameroon’s Anglophone crisis. https://www.bbc.com/news/world-africa-45723211.

[20] Kaiser, David. E. Economic Diplomacy and the Origins of the Second World War. Princeton: Princeton University Press; 2015. 10.1515/9781400875719.

[21] Minsanté-DRH. Analyse de la situation des ressources humaines pour la santé. Yaoundé: Ministere de la Sante Publique, Cameroun; 2010.

[22] Programmeme Elargi De Vaccination (2018). Normes et Standards.

[23] Asobie A (2003). Nigeria, Cameroun and the unending conflict over Bakassi.

[24] International Crisis Group. Cameroon’s Anglophone Dialogue: A Work in Progress (2019). Available from: https://www.crisisgroup.org/africa/central-africa/cameroon (Accessed 30 Dec 2019), *I*.

[25] United Nations High Commission on Refugies. Cameroon situation, responding to the needs of idps and cameroonian refugees in nigeria, Supplementary Appeal, January–December 2019. Available from: http://reporting.unhcr.org/sites/default/files/UNHCR%20Came. Accessed 24 Jan 2019.

[26] Calvin T (2019). Routine Immunisation Performance in Cameroon.

[27] Biggs GJ, Ann B (2019). The impact of conflict on immunisation coverage in 16 countries. Int J Health Policy Manag.

[28] Ziad M, Randa H, Alissar R, Carolina D-HM, Kamal F, Racha S, Lina B, Ramy W, Walid A (2019). Vaccination coverage in Lebanon following the Syrian crisis: Results from the district-based immunisation coverage ev. BMC Public Health.

[29] Torbosh A, Alemad MA, Al-Serouri A, Khader Y (2019). The impact of war in Yemen on under one year children immunisation coverage (Preprint). JMIR Public Heal Surveill.

[30] Paul A (2019). Hospital attack in anglophone Cameroon kills four patients. Lancet (London, England).

[31] Pariyo AH, William G (2013). Violence against health workers during armed conflict. Lancet.

[32] World Health Organisation. World Health Day 2009—save lives. Make hospitals safe in emergencies. http://www.who.int/hac/events/7april2009/en/index.html (Accessed 29 Nov 2019).

